# Histone-DNA Complexes and Coagulation after Intracerebral and Subarachnoid Hemorrhage

**DOI:** 10.1055/s-0041-1728672

**Published:** 2021-04-14

**Authors:** Tua Gyldenholm, Anne-Mette Hvas, Signe V. Lauridsen, Emilie Sandgaard, Christine L. Hvas

**Affiliations:** 1 Thrombosis and Hemostasis Research Unit, Department of Clinical Biochemistry, Aarhus University Hospital, Aarhus, Denmark; 2 Department of Clinical Medicine, Aarhus University, Aarhus, Denmark; 3 Department of Anesthesiology and Intensive Care, Aarhus University Hospital, Aarhus, Denmark

## Introduction

Intracerebral hemorrhage (ICH) and subarachnoid hemorrhage (SAH) have limited treatment options and a high mortality. We have previously shown that both conditions are associated with early hypercoagulation. 1 2

Histones have been demonstrated to promote thrombin generation. 3 In sepsis and trauma patients, studies have revealed an association between histone-DNA complexes and free histone levels and mortality or morbidity. 4 5 6 Theoretically, the effects on coagulation of histones can be investigated in vitro by attempting to block histones with specific antibodies.

The aim of this study was to quantify histone-DNA complex levels in patients with ICH or SAH at the time of admission to hospital compared with 24 hours after symptom onset and to investigate the possible in vitro effect of anti-histone antibodies on thrombin generation.

## Materials and Methods

The study was a prospective cohort study. Inclusion criteria were radiologically verified spontaneous ICH or SAH. Patients were excluded if they received medications affecting coagulation prior to inclusion. Further details on the cohort and exclusion criteria have been previously published. 1 2 Blood samples were drawn from a peripheral vein or arterial line at admission to hospital and 24 ± 2 hours after symptom onset. Samples were batch analyzed shortly after patient inclusion ended.

Histone-DNA complexes were detected by using the Cell-Death Detection ELISA ^PLUS^ kit (Roche, Mannheim, Germany) and reported in absorbance units (AU). A cohort of 20 healthy men and 20 healthy women established the control group for histone-DNA complexes. 7

Thrombin generation was measured in platelet-poor plasma with the addition of tissue factor (5 pm ), phospholipids (4 µM), FluCa, hepes, calcium, murine IgG2a kappa isotype control antibody (StemCell Technologies, Vancouver, Canada) and anti-histone antibodies MHIS AB1952 (specific for histone H4) and MHIS AB1992 (specific for histones H1 and H3) kindly donated by Charles T. Esmon, Oklahoma Medical Research Foundation, Oklahoma City, United States. An antibody concentration equivalent to 0.426 mg/mL was chosen for thrombin generation analyses after titration experiments based on available murine studies. 8 Four analyses were performed per sample: one with addition of NaCl, one with addition of control antibody and two with the addition of AB1952 and AB1992, respectively. Thrombin generation was quantified with calibrated automated thrombogram (CT, thrombinoscope BV, Maastricht, Holland). A cohort of 45 healthy men and 45 healthy women established a control group for thrombin generation results. 9

Furthermore, international normalized ratio (INR), activated partial thrombin time (aPTT), antithrombin (functional), thrombin time, fibrinogen (functional, Clauss method), fibrin D-dimer, hemoglobin, platelet and leucocyte count, C-reactive protein (CRP), alanine transaminase, albumin, creatinine, and S100 Calcium Binding Protein B (S100B) were determined.

Descriptive statistics are presented as median with interquartile range (IQR). Paired data were analyzed with a Wilcoxon's signed rank test (not following Gaussian distribution) or with a Student's *t* -test (following Gaussian distribution). Unpaired data were analyzed with a Mann–Whitney test. Correlation tests were performed by using a Spearman's test. Sample size was defined by the parent study. The primary endpoint was the change in histone-DNA complex levels from admission to 24 hours after symptom onset, for which the power was 100% (mean value 17 AU at admission [standard deviation 16 AU] and 6 AU at 24 hours) and the significance level, 2α, was 0.05).

The study was conducted in accordance with the Declaration of Helsinki, and the Central Denmark Region Committees on Health Research Ethics approved the study before initiation (case no. 1–10–72–95–14).

## Results and Discussion

Eighty-seven patients with ICH or SAH were included. The median age was 61 years, and 67% were females. Of the 46 patients with an SAH, 17 underwent surgical coiling and thus received unfractionated heparin. Overall 30-day mortality was 26%, with 33% mortality for SAH patients, and 20% mortality for ICH patients.

Table 1 shows the standard biochemical profiles at admission and 24 hours after symptom onset. Seven patients had a prolonged thrombin time 24 hours after symptom onset due to administration of unfractionated heparin during the coil procedure, close to sample collection. Exclusion of these patients had no significant impact on the results described below.

**Table 1:** Biochemical values at time of admission to hospital and 24 hours after symptom onset in 87 patients with intracerebral or subarachnoid hemorrhage

Parameter	Reference values a	At admission	After 24 hours	*p* -Values
Hemoglobin (mmol/L) ( *n*_paired_ = 73)	7.3–9.5	8.4 (7.7–8.9)	7.4 (6.9–8.3)	<0.0001
Platelet count (× 10 ^9^ /L) ( *n*_paired_ = 72)	165–400	229 (179–268)	212 (170–251)	0.003
INR ( *n*_paired_ = 71)	<1.2	1.0 (1.0–1.1)	1.1 (1.1–1.2)	<0.0001
aPTT (s) Before April 5, 2016 ( *n*_paired_ = 68)	25–38	28 (26–31)	31 (28–34)	<0.0001
aPTT (s) After April 5, 2016 ( *n*_paired_ = 9)	20–29	27 (25–29)	25 (24–31)	0.88
Antithrombin (× 10 ^3^ IU/L) ( *n*_paired_ = 37)	0.80–1.20	0.92 (0.86–1.04)	0.92 (0.85–1.01)	0.84
Thrombin time (s) ( *n*_paired_ = 57)	<21	16 (16–17)	16 (15–17)	0.64
Fibrinogen (μmol/L) ( *n*_paired_ = 71)	5.0–12.0	9.2 (7.8–11.1)	9.8 (8.3–10.8)	0.004
Fibrin d-dimer (mg/L FEU) ( *n*_paired_ = 71)	<0.5	1.3 (0.66–2.20)	1.2 (0.6–3.5)	0.29
S100B (μg/L) ( *n*_paired_ = 71)	0.02–0.13	0.13 (0.07–0.41)	0.13 (0.06–0.24)	0.33
CRP (mg/L) ( *n*_paired_ = 73)	< 8.0	2.2 (0.9–4.5)	11.9 (3.9–37.8)	<0.0001
Leucocytes (× 10 ^9^ /L) ( *n*_values_ = 78)	3.5–10.0	10.9 (7.7–13.4)		
Alanine Transaminase U/L ( *n*_values_ = 84)	10–70	20 (16–30)		
Albumin (g/L) ( *n*_values_ = 86)	36–45	38 (35–40)		
Creatinine (μmol/L) ( *n*_values_ = 86)	45–105	60 (53–75)		

Abbreviations: aPTT, activated partial thrombin time. CRP, C-reactive protein; INR, international normalized ratio; S100B, serum s100 calcium binding protein B.

Note: Values reported as median (interquartile range). As there are missing values (due to failed blood sampling, patient dying before blood sampling etc.), we have reported *n*_paired_ = number of paired values, *n* values = number of values. The reference interval for aPTT changed during the inclusion period, and aPTT data are split in two groups accordingly.

a Reference values for adults, combined for men and women, established at the local laboratory.

ICH patients and SAH patients did not differ in histone-DNA complex levels measured at admission ( *p* = 0.34) or 24 hours after symptom onset ( *p* = 0.13). For the total cohort, histone-DNA complex levels decreased from a median = 11.6 AU (IQR = 6.5–24.5) at admission to median = 3.5 AU (1.5–8.2) 24 hours after symptom onset ( *p* < 0.0001). Patients had significantly higher levels of histone-DNA complexes at admission compared with the healthy cohort mean = 5.4 AU (2.0–11.4; *p* = 0.0001).

No difference was demonstrated in histone-DNA complex levels at admission for 30-day survivors (median = 11.3 AU (IQR = 6.4–23.2) compared with nonsurvivors (11.4 AU [5.1–25.9], *p* = 1.00). That histone-DNA complex levels were not found to correlate with mortality is in accordance with a study performed on trauma patients. 10 Thus, we could not confirm the association between levels of histone-DNA complexes and morbidity and mortality found in other patient populations. 4 5 6 These differences between studies could be due to the size of our study or to the only moderate levels of histone-DNA complexes found in the present study.

**Fig. 1:**
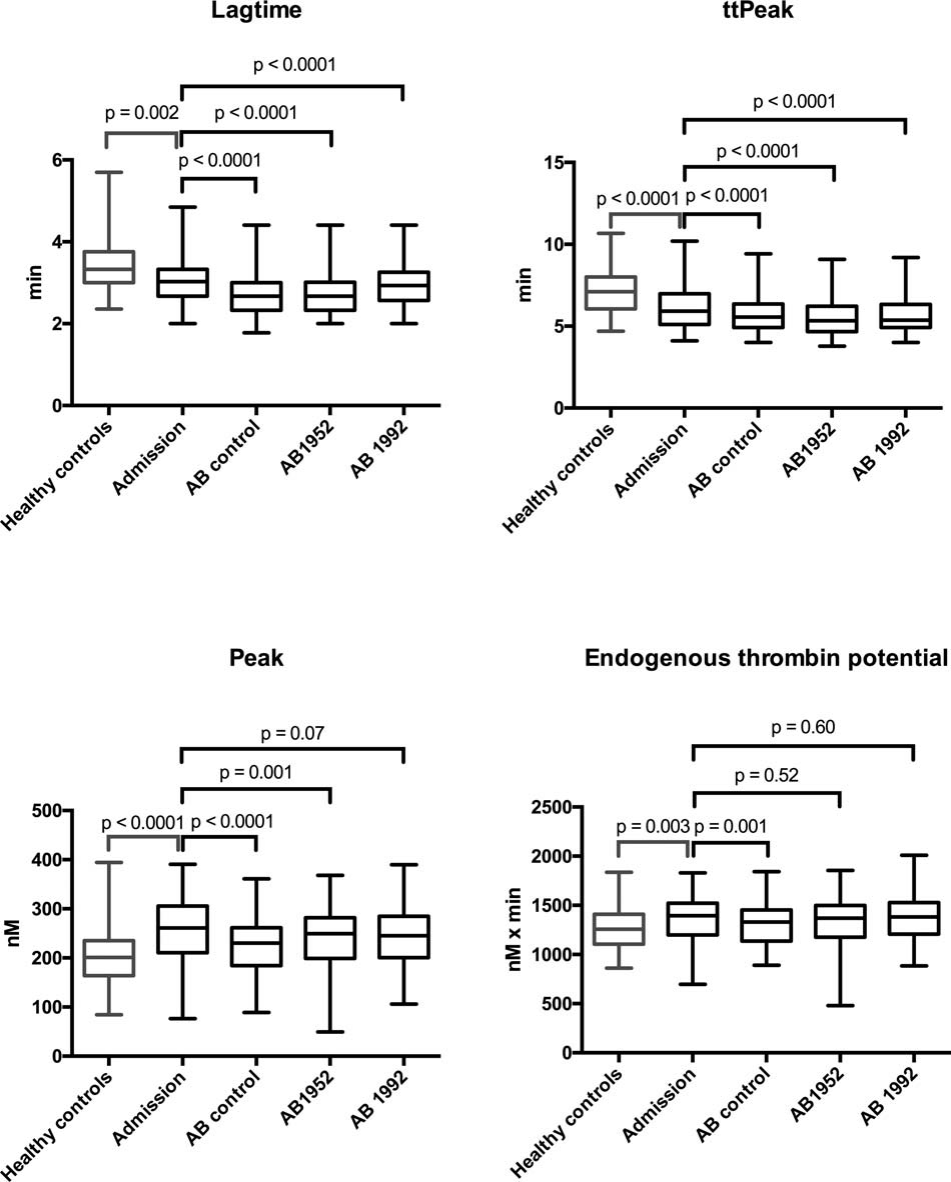
Thrombin generation with antibody addition in 87 patients with intracerebral or subarachnoid hemorrhage at admission. Thrombin generation in healthy controls and in patients with an intracerebral or subarachnoid hemorrhage, described by lagtime, time to peak, peak and endogenous thrombin potential, with and without added control antibody and anti-histone AB1952 and AB1992 antibodies. Boxes and whiskers indicate median, interquartile range, and range. Number of measurements per group is 81 due to missing values in six patients.

Fig. 1 shows thrombin generation performed on healthy controls as well as patient admission samples with and without added control and anti-histone antibodies. The patient admission samples had significantly decreased lagtime and time to peak and increased peak and endogenous thrombin potential (ETP; all *p* ≤ 0.003) compared with the healthy controls, signifying an increased thrombin generation.

The anti-histone antibodies reduced time to peak when compared with the control antibody ( *p* < 0.0001 for AB1952 and *p* = 0.05 for AB1992). However, the addition of anti-histone antibodies did not suppress thrombin generation more than the control antibody for ETP, peak, and lagtime values. Thus, the suppression may be due to a physical effect of adding the antibody molecules, regardless of their specificity. Furthermore, the antibodies only block histones and not the entire histone-DNA complex.

The main finding of the present study was increased levels of histone-DNA complexes in the acute phase right after an ICH or SAH when compared with 24 hours after symptom onset. A higher thrombin generation was demonstrated in patients at admission than in healthy controls. No convincing effect of adding anti-histone antibodies to thrombin generation could be discerned. Hence, the present study did not support the hypothesis that histones have a significant effect on coagulation as expressed by thrombin generation.
